# Terpenoids From Cannabis Do Not Mediate an Entourage Effect by Acting at Cannabinoid Receptors

**DOI:** 10.3389/fphar.2020.00359

**Published:** 2020-03-25

**Authors:** David B. Finlay, Kathleen J. Sircombe, Mhairi Nimick, Callum Jones, Michelle Glass

**Affiliations:** ^1^Department of Pharmacology and Toxicology, University of Otago, Dunedin, New Zealand; ^2^Soma Group, Dunedin, New Zealand

**Keywords:** cannabis, cannabinoid, terpenoid, terpene, entourage effect, signaling, binding

## Abstract

The entourage effect was a proposed explanation for biological observations that endocannabinoid ligand activities can be modified by other lipids released from cells at the same time. An increasing volume of anecdotal reports and interest in the plant have provoked research into the activity of minor chemical constituents of the plant—including volatile terpenoids such as myrcene, α- and β- pinene, β-caryophyllene, and limonene. However, to date, no clear interaction has been identified. The current study was designed to determine whether terpenes in the cannabis plant have detectable receptor-mediated activity, or modify the activity of Δ^9^-tetrahydrocannabinol, cannabidiol, or the endocannabinoid 2-arachidonylglycerol at the cannabinoid receptors. In addition, we have utilized a standard radioligand binding paradigm with ability to detect orthosteric and allosteric interactions of test compounds. With the possible exception of a weak interaction of β-caryophyllene with CB2, no data were produced to support the hypothesis that any of the five terpenes tested (either alone or in mixtures) have direct interactions with CB1 or CB2, as the binding of radioligand ([^3^H]-CP55,940), Δ^9^-tetrahydrocannabinol, and cannabidiol were unaltered by the presence of terpenes. Similarly, terpene functional effects were also not detected, either alone or in combination with Δ^9^-tetrahydrocannabinol, cannabidiol, or 2-arachidonoylglycerol. This study adds to the evidence that the putative entourage effect cannot be explained by direct effects at CB1 or CB2.

## Introduction

Cannabinol (CBN) was the first cannabinoid from the cannabis plant for which a structure was identified (Cahn, 1933). Cannabidiol (CBD) was identified a few years later (Adams et al., 1940a), and the same research group later came close to identifying the structure of tetrahydrocannabinols, in a study involving isomerization of CBD (Adams et al., 1940b). Shortly after, tetrahydrocannabinols were isolated from cannabis resin (Wollner et al., 1942)—though it was more than 20 years before chemical analytical methods were adequate for resolving the final structure of the main psychoactive component of cannabis, (−)Δ^9^-tetrahydrocannabinol (Δ^9^-THC; Gaoni and Mechoulam, 1964).

Meanwhile, Loewe was also the first to observe pharmacological differences between cannabinoids (Loewe, 1946), in a study differentiating Δ^9^-THC and a synthetic hexyl analog, from CBD: the former, but not the latter, caused catalepsy and central excitation (with some additional species differences). In the years since, at least 489 different compounds (ElSohly and Slade, 2005), including at least 113 cannabinoids (Aizpurua-Olaizola et al., 2016), have been identified from cannabis. The most abundant of these are Δ^9^-THC and CBD (Aizpurua-Olaizola et al., 2016; Scherma et al., 2018). Δ^9^-THC acts as a partial agonist at type 1 cannabinoid receptors (CB1), which are found mostly in the central and peripheral nervous system and mediate the intoxicating effects for which cannabis is well known (reviewed in Pertwee, 2008a; Pertwee, 2008b). It also acts at type 2 cannabinoid receptors (CB2), which are most highly expressed in immune cells (reviewed in Turcotte et al., 2016). In general, many of the effects of CBD are thought to occur through non-cannabinoid receptor mechanisms (Turner et al., 2017). However, CBD has been demonstrated to bind to CB2 at high (micromolar) concentrations (Pertwee, 2008b)—although this is also controversial, as some evidence suggests that at much lower concentrations than this, CBD may behave as an inverse agonist at CB2 and an antagonist (Thomas et al., 2007) or allosteric modulator (Laprairie et al., 2015) of CB1.

More recently, interest has also turned to the biological activity of the less abundant, “minor” phytocannabinoids and phytoterpenoids, and their ability to produce an “entourage effect”. This phenomenon was first described for endogenous glycerol esters (Ben-Shabat et al., 1998), when 2-linoleoylglycerol and 2-palmitoylglycerol were found to increase the on-target affinity and efficacy of the endogenous cannabinoid 2-arachidonoylglycerol (2-AG), with which they co-occur, in spleen—yet without detectable direct interaction with the cannabinoid receptors themselves (though these data were not shown). Similar observations have been described for *N*-palmitoylethanolamide and *N*-oleoylethanolamide (which are co-synthesized with anandamide) and may potentiate anandamide-induced relaxation of arteries (Ho et al., 2008).

Since the publication of the Ben-Shabat et al. study, the term “entourage effect” has been co-opted to refer to the idea that whole cannabis possesses greater therapeutic potential than its individual components (Russo, 2011; Worth, 2019), with many websites suggesting that terpenes can modify the high produced by Δ^9^-THC (e.g., https://www.heylocannabis.com/post/what-are-terpenes). Terpenoids are commonly found in plants (Gershenzon and Dudareva, 2007), and at least 120 have been found in cannabis (ElSohly and Slade, 2005)—of which some of the most commonly referenced appear to include linalool, myrcene, limonene, β-caryophyllene, and α- and β-pinene. Previous work has suggested that β-caryophyllene may act as a CB2 agonist (Gertsch et al., 2008), though subsequent studies have questioned this (Santiago et al., 2019).

Evidence for cannabis-derived terpenoids having entourage activity is also sparse. A very recent study has attempted to examine the six terpenoids referred to above for potential entourage activity at cannabinoid receptors. When used either alone or in combination to stimulate AtT-20 cells expressing CB1 or CB2, Δ^9^-THC-induced hyperpolarization was unaffected (Santiago et al., 2019)—indeed no GIRK channel-related modulatory effects were detected in this molecular study for any of the terpenes. In a related GIRK assay paradigm, receptor desensitization was also unaffected (Santiago et al., 2019).

The current study aimed to clarify the putative molecular activity of five terpenoids of interest acting specifically (on-target) through CB1/CB2, in a canonical activity pathway (cAMP) which can capture receptor effects with high sensitivity. Effects on orthosteric ligand binding were also included in the study design, as in addition to detecting orthosteric interactions this assay has been shown to be very sensitive to allosteric modulation of CB1 (Ahn et al., 2012; Ignatowska-Jankowska et al., 2015).

## Materials and Methods

### Drugs

All terpenes were purchased from True Terpenes (Portland, OR). Terpene molarities were calculated from the density and purity specified on the supplier’s technical data sheets (**Table 1**). Terpenes were diluted to 10 mM in DMSO (Sigma Aldrich, St Louis, MO, USA), and DMSO content was kept consistent in all assays at 1:1,000. Terpenes were assessed in assays separately, and in three different mixtures (**Table 2**): commercial analysis of multiple cannabis variants indicate huge variability in terpenoid formulations between strains (*e.g.*, www.weedmd.com/terpene-profiles), and these mixtures were therefore intended to capture some of this variability.

**Table 1:** Terpene purity specifications and calculated molarity (True Terpenes, OR, USA).

Terpene	Density (g/ml)	Purity	Concentration (M)
Myrcene	0.794	97.6%	5.69
α-Pinene	0.859	99.3%	6.26
β-Pinene	0.860	98.2%	6.20
β-Caryophyllene	0.908	91.0%	4.04
Limonene	0.841	99.1%	6.12

**Table 2:** Constitution of terpene mixtures.

Terpene	Mixture 1 (%)	Mixture 2 (%)	Mixture 3 (%)
Myrcene	40	30	50
α-Pinene	20	17	23
β-Pinene	15	13	17
β-Caryophyllene	20	35	5
Limonene	5	5	5
**Total**	**100**	**100**	**100**

Δ^9^-THC was purchased as resin from THC Pharma GmbH (Frankfurt, Germany), CBD was purchased from Tocris (Bristol, UK), and 2-AG was purchased from Cayman Chemical Company (Ann Arbour, MI). Each was constituted in absolute ethanol at 31.6 mM, and diluted (in vehicle) as required so that the final ethanol content in assays was 1:1,000. Forskolin was purchased from Cayman Chemical Company, and prepared in DMSO at 31.6 mM. All compounds were ≥98% purity, with the exception of THC which was ≥ 95%.

### Radioligand Binding Assays

Competition displacement radioligand binding assays were performed as previously described (Finlay et al., 2017). In brief, HEK cells expressing either human CB1 receptors N-terminally tagged with preprolactin signal sequence (pplss) and 3x haemagglutinin (3HA) epitopes (Finlay et al., 2017) or human CB2 receptor N-terminally tagged with 3HA (Grimsey et al., 2011) were harvested in 5 mM EDTA in PBS, and “P2” membranes were prepared in sucrose buffer as previously described (Finlay et al., 2017). Protein content was estimated using a BioRad (Hercules, CA) DC protein assay (modified Lowry assay). For binding assays, radioligand ([^3^H]-CP55,490, PerkinElmer, Waltham, MA, USA), non-radiolabelled drugs, and P2 membrane preparations were diluted in binding buffer (50 mM HEPES pH 7.4, 1 mM MgCl_2_, 1 mM CaCl_2_, 2 mg/ml NZ-origin BSA, MP Biomedicals, Santa Ana, CA, USA) and dispensed into 96‐well, polypropylene V‐well plates (Hangzhou Gene Era Biotech Co Ltd, Zhejiang, China) in a final reaction volume of 200 µl (membranes were dispensed last). Final radioligand concentration was 1 nM, and protein content was 3 µg/point for pplss-3HA-hCB1 HEK membranes, and 2 µg/point for 3HA-hCB2 HEK membranes.

When all components had been dispensed, the plate was sealed and incubated for 1 h at 30°C. During the incubation, a 96 well harvest plate (GF/C filters, 1.2 µm pores) was treated with 0.1% w/v branched polyethyleneimine (PEI; Sigma Aldrich) in water. Immediately prior to washing, PEI was washed through the filters using a vacuum manifold (Pall Corporation, Port Washington, NY) and all wells were washed once with ice cold wash buffer (50 mM HEPES pH 7.4, 500 mM NaCl, 1 mg/ml BSA. Equilibrated binding mixture was then transferred to the harvest plate under vacuum, and samples washed through. Binding wells were rinsed once with wash buffer and transferred to the harvest plate, and then wells were washed three more times with 200 µl of wash buffer. The plate was then removed, and filters allowed to dry overnight.

The next day, the plate bottom was sealed, and 50 µl of Ultima Gold XR scintillation fluid (PerkinElmer) was dispensed to each well. The plate top was then sealed, and the plate was loaded into a 96 well “rigid” cassette and loaded into a Wallac MicroBeta2^®^ TriLux Liquid Scintillation Counter (PerkinElmer). Scintillation was detected after a 30 min delay, for 2 mins per well. Counts were corrected for detector efficiency. Data were then exported and analyzed in GraphPad Prism v8 (GraphPad Software Inc., La Jolla, CA, USA), and presented normalized to total binding ([^3^H]-CP55,940 alone; 100%), and maximum displacement (binding in the presence of 10 µM Δ^9^-THC).

### Functional Assay: Cyclic AMP Signaling

Cellular cAMP was measured using a commercially available BRET assay (CAMYEL), as previously described (Jiang et al., 2007; Cawston et al., 2013). In brief, HEK cells expressing either N-terminally tagged 3HA-tagged hCB1 (first reported in Cawston et al., 2013) or HA-3TCS-hCB2 (first reported in Cawston et al., 2015) were seeded in 10 cm cell culture dishes, and cultured overnight in high glucose DMEM (Hyclone, GE Healthcare, Chicago, IL) supplemented with 10% fetal bovine serum. Cells were then 40–60% confluent, and were transfected with pcDNA3L-His-CAMYEL encoding the CAMYEL biosensor (cAMP sensor with YFP-Epac-RLuc). Transfection was performed by combining 30 µg linear PEI (Polysciences, Warrington, PA, USA) from stock at 1 mg/ml, with 5 µg of CAMYEL plasmid, in a total volume of 500 µl of 150 mM sterile NaCl. Transfection mixture was incubated for 10 mins, then culture medium was replaced and the transfection mixture was dispensed. Dishes were returned to the incubator and cultured overnight. Cells were then lifted with 0.05% trypsin/EDTA (Gibco Thermo Fisher Scientific, Waltham, MA, USA), and seeded at high density (60,000 cells per well) in white 96 well CulturPlates (PerkinElmer) which had been pre-treated with 0.05 mg/ml high molecular weight poly-D-lysine (Sigma) in PBS, to increase adherence.

On assay day, well contents were aspirated with a strip vacuum (Integra Biosciences, Hudson, NH, USA), and wells were washed once with PBS to remove traces of phenol red. Wells were serum starved for 35 mins prior to stimulation in “assay medium”—phenol-free, high glucose DMEM (Hyclone) supplemented with 1 mg/ml BSA and 10 mM HEPES pH 7.4 (Gibco, Thermo). Drugs were prepared at 10x concentration during serum starvation. Five minutes prior to stimulation, Rluc substrate coelenterazine-H (Prolume, Pinetop, AZ, USA; prepared as 5 mM stock in absolute ethanol) was dispensed to the wells to be assayed (final concentration 5 µM). Forskolin, cannabinoid agonists, CBD, and terpenes (or their vehicles, as relevant) were each prepared and mixed together in a dispensing plate. At the start of the assay run, drugs were transferred into assay wells with a multichannel and immediately loaded into a pre-warmed (37°C) plate reader. CAMYEL biosensor emission signals were detected in a LUMIstar Omega plate reader (BMG Labtech, Ortenberg, Germany), using simultaneous detection BRET1 filters (475/30 and 535/30 nm) over a period of approximately 20 mins.

Inverse BRET ratios (460/535 nm) were plotted in GraphPad Prism against time. These data were analyzed by “area under the curve” (AUC) and normalized to a matched basal (vehicle alone, 0%) and 5 µM forskolin (100%) conditions. All terpenes, 2-AG, and CBD were applied at concentrations of 10 µM, Δ^9^-THC was used at a concentration of 1 µM. Each individual experiment was carried out in duplicate and repeated at least three times.

### Statistics

**Figure 1:**
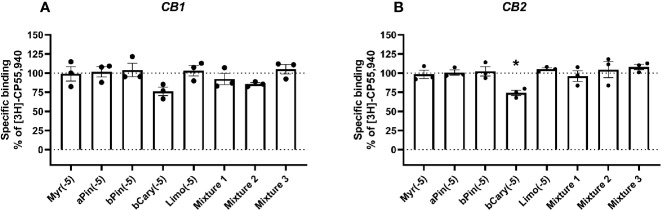
Specific binding of [3H]-CP55,940, with displacement by terpenes (10 µM) in membranes containing hCB1 **(A)** or hCB2 **(B)**. Binding data in all plots are normalized to total [3H]-CP55,940 binding in the absence of displacer (100%), and in the presence of 10 µM THC (0%). Data are means ± SEM of three independent determinations. *p < 0.05.

**Figure 2:**
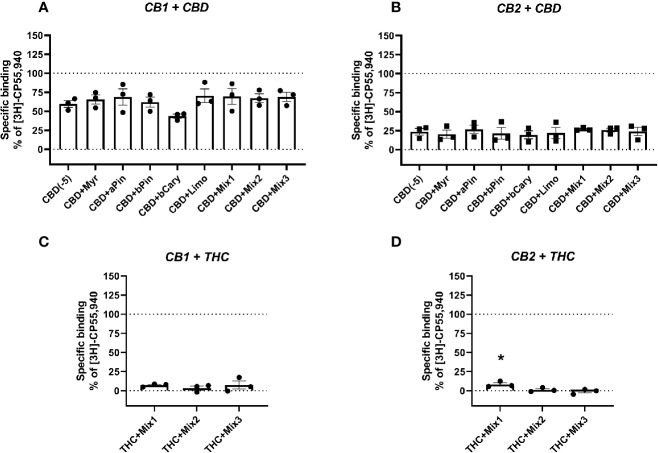
Specific binding of [^3^H]-CP55,940, with displacement by CBD **(A, B)** or THC **(C, D)** in the presence and absence of terpenes (all at 10 µM) in membranes containing hCB1 **(A, C)** or hCB2 **(B, D)**. Binding data are normalized to total [3H]-CP55,940 binding in the absence of displacer (100%), and in the presence of 10 µM THC (0%). Data are means ± SEM of three independent determinations. *p < 0.05.

All statistical tests were performed in GraphPad Prism v8, and entailed 1-way ANOVAs followed by Holm-Šídák tests when tested means were found to be statistically significantly different overall. Tests were run separately for each receptor. For binding data, tests were performed for total binding versus each terpene alone, and when terpenes were screened in combination with other drugs (Δ^9^-THC or CBD) then tests were performed for each matched condition (i.e., Δ^9^-THC+terpene was compared to Δ^9^-THC; CBD+terpene was compared to CBD). Significant differences in figures of binding data (**Figures 1** and **2**) are denoted by an asterisk (*, p < 0.05).

**Figure 3:**
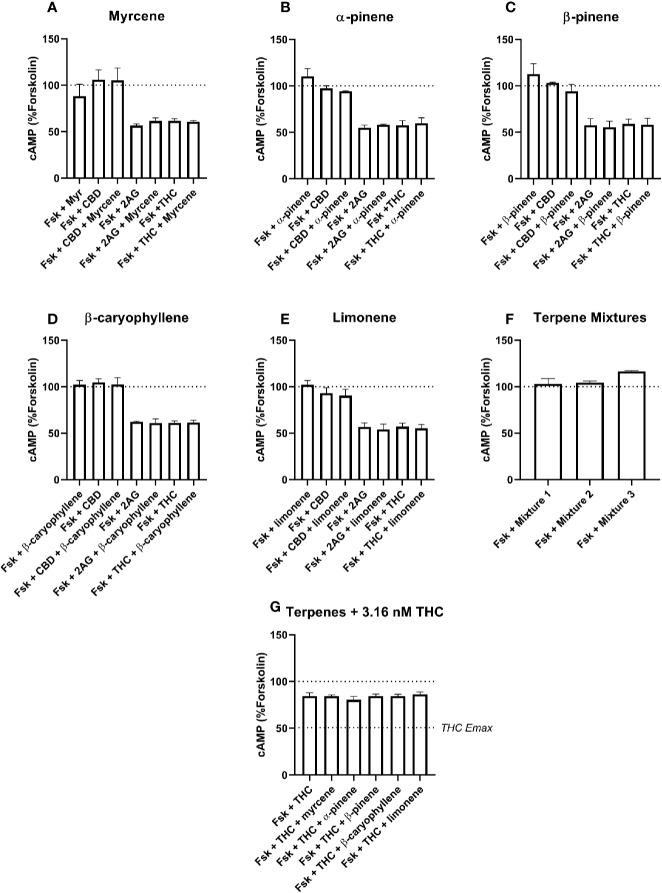
hCB1-Mediated inhibition of cAMP production in response to forskolin (Fsk) and drug combinations with 10 µM myrcene **(A)**, α-pinene **(B)**, β-pinene **(C)**, β-caryophyllene **(D)**, limonene **(E)**, or terpene mixtures **(F)**. Figure **(G)** shows inhibition of the Fsk response by an approx. EC50 concentration of THC (3.16 nM) in combination with 10 µM of each of the five terpenes. Data are normalized to forskolin (100%) and basal (0%). cAMP responses were stimulated with forskolin (5 µM), and all cannabinoids and terpenes were at a final concentration of 10 µM. Data are means ± SEM of three independent determinations.

**Figure 4:**
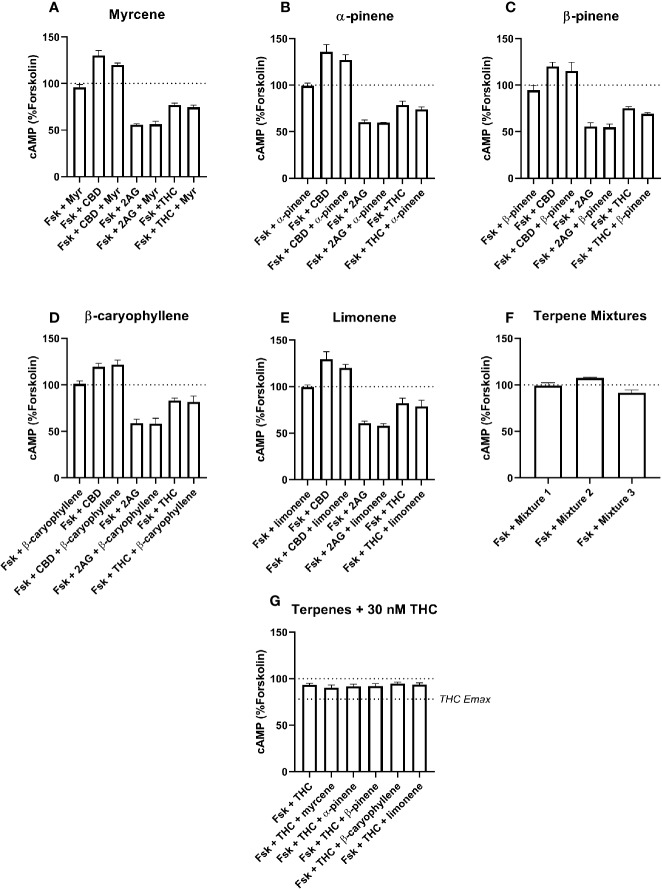
hCB2-Mediated inhibition of cAMP production in response to forskolin (Fsk) and drug combinations with 10 µM myrcene **(A)**, α-pinene **(B)**, β-pinene **(C)**, β-caryophyllene **(D)**, limonene **(E)**, or terpene mixtures **(F)**. Figure **(G)** shows inhibition of the Fsk response by an approx. EC50 concentration of THC (30 nM) in combination with 10 µM of each of the five terpenes. Data are normalized to forskolin (100%) and basal (0%). cAMP responses were stimulated with forskolin (5 µM), and all cannabinoids and terpenes were at a final concentration of 10 µM. Data are means ± SEM of three independent determinations.

For cAMP data, tests were performed for forskolin alone, and with each other drug (2-AG, Δ^9^-THC, CBD, terpene, or terpene mixture). Separate tests were performed for effects in assays involving drug combinations. In these cases, post-testing was performed to compare paired matches of conditions with and without terpenes (*e.g.* Fsk+CBD was compared with Fsk+CBD+Myrcene, *etc*.). Note that statistical significance for cAMP data are not shown in **Figure 3** or **4**—this was in order to avoid confusion about which pairs of conditions were significantly different. Instead, important results are referred to in-text.

## Results

### Radioligand Binding Assays

At concentrations of 10 µM (given in **Figures 1** and **2** as log molar, −5), none of the terpenes tested significantly altered the binding of [^3^H]-CP55,940 in membranes containing CB1 (**Figure 1A**). Similarly, in CB2-containing membranes (**Figure 1B**), four of the five terpenes alone did not alter radioligand binding. The exception to this was β-caryophyllene, which displaced [^3^H]-CP55,940 to a modest extent (approximately 25% of specific binding). No condition altered binding sufficiently to justify a full curve.

To test an entourage-related concept that terpenes may act by modifying the binding of other ligands (particularly those also from the cannabis plant, CBD and Δ^9^-THC), the terpenes and/or terpene mixtures (**Table 2**) were tested for their ability to alter displacement of the radioligand by both of these drugs. CBD displaced the radioligand in both CB1- and CB2-containing membranes, as expected (reviewed in Pertwee, 2008b)—[^3^H]-CP55,940 binding decreased to mean 59.70% and 20.26% of specific binding, respectively. However, no terpene or terpene mixture significantly altered CBD displacement of the radioligand in either membrane (**Figures 2A, B**). Similarly, no significant difference in displacement of the radioligand by Δ^9^-THC was induced by terpene mixtures for CB1 (**Figure 2C**), while at CB2 (**Figure 2D**) the combination of Δ^9^-THC with mixture 1 slightly but significantly decreased displacement ([^3^H]-CP55,940 binding was increased from 0% to 8.26% of the window, reflecting a small reduction in displacement by Δ^9^-THC).

### Functional Assay: Cyclic AMP Signaling

Signaling responses to the known cannabinoid agonists 2-AG and Δ^9^-THC were as expected; both significantly inhibited cAMP production induced by 5 µM forskolin at both CB1 (**Figure 3**, p < 0.05) and CB2 (**Figure 4**, p < 0.05). As these agonists were included as matched controls in assays for each of the five terpenes tested, five determinations were obtained for each 2-AG and Δ^9^-THC in each of three independent assay replicates (n=15). At CB1, mean inhibition of the forskolin response by 2-AG and Δ^9^-THC was 42.4% (± 1.7%) and 40.7% (± 1.6%), respectively (**Figures 3A–F**). Full concentration-response curves were performed for Δ^9^-THC in the CB1 cell line, producing a mean pEC50 of 8.50 (± SEM 0.05, n=3).

In the CB2 cell line, the extents of inhibition differed more, with 2-AG driving 41.7% (± 1.3%) inhibition of the forskolin response, but Δ^9^-THC appearing much lower efficacy—just 20.5% (± 1.6%) of the forskolin response was inhibited (**Figures 4A–F**). The Δ^9^-THC pEC50 determined in the CB2 cell line was 7.80 (± SEM 0.06, n=3). The effects of CBD at CB1 and CB2 also differed, having no significant effect at CB1, but acting as an inverse agonist at CB2, consistent with a previous report (Thomas et al., 2007), here driving a significant increase in cAMP of 27.0% (± 2.9%) above forskolin alone.

None of the five terpenes screened, either alone or in mixtures, modified cAMP signaling significantly through either CB1 (**Figure 3**) or CB2 (**Figure 4**). Statistical tests to determine this were performed by a single 1-way ANOVA for each cell line, using multiple comparisons to allow comparisons of paired conditions—i.e., each orthosteric ligand with terpene *versus* a matched condition in absence of terpene.

An additional Δ^9^-THC condition was also included, to determine whether 10 µM of any terpene would modify the cAMP signaling of an approx. EC50 concentration of Δ^9^-THC at either CB1 (3.16 nM) or CB2 (30 nM). The purpose of this condition was to capture terpene-induced alterations to the potency of the Δ^9^-THC response in each cell line. However, consistent with the data at higher Δ^9^-THC concentrations, no change in cAMP signaling was observed in the presence of any of the five terpenes at either CB1 (**Figure 3G**) or CB2 (**Figure 4G**). For results from both CB1 and CB2 cell lines, 1-way ANOVAs were performed but no differences in means were found in the tested conditions.

## Discussion

Overall, these data do not support the idea that any of the five terpenes tested in this study contribute to a putative entourage effect directly through the cannabinoid receptors. β-Caryophyllene was found to bind weakly to CB2 alone, but no other functional or binding effects were detected for the terpenes alone or in combination with CBD, or cannabinoid agonists 2-AG and Δ^9^-THC. CBD is increasingly becoming a focus of therapeutic studies due to positive results in a series of childhood epilepsy clinical trials (Devinsky et al., 2017; Laux et al., 2019), yet its mechanism of action remains unclear, with over 65 putative molecular targets identified (Bih et al., 2015). We were therefore interested to investigate whether the terpenes could enhance its activity or affinity for cannabinoid receptors, providing a mechanism for interaction with the endocannabinoid system. In this study we confirmed low affinity interactions with CB1 and CB2, as previously reported (reviewed in Pertwee, 2008b). The extent of displacement observed in this study (at 10 µM concentrations) are consistent with Ki values in the low micromolar range reported for CB2, and >10 µM for CB1 (McPartland et al., 2007). In the cAMP assay, CBD showed inverse agonist activity at CB2, again consistent with previous studies (Thomas et al., 2007), but no activity was detected at CB1. The terpenes did not modify either the binding or the functional response of CBD at either receptor.

Radioligand binding experiments can detect both direct orthosteric interactions with a receptor, and in many cases, (including for CB1) allosteric modulation (Ahn et al., 2012; Ignatowska-Jankowska et al., 2015). The assay design used here provides detection of displacement (such as observed for the orthosteric ligands, 2-AG and Δ^9^-THC; **Figures 1** and **2**) or enhancement of binding (as seen for all current positive and negative allosteric modulators of CB1, Price et al., 2005; Ahn et al., 2012; Ignatowska-Jankowska et al., 2015). In this light, the lack of binding modulation by the terpenes (excluding β-caryophyllene at CB2) suggests a lack of both orthosteric and allosteric modulation of binding. Significant alterations in radioligand binding by a terpenoid were detected for β-caryophyllene alone (which significantly displaced the radioligand at CB2, **Figure 1B**) and the combination of 10 µM Δ^9^-THC with mixture 1, also at CB2 (**Figure 2D**), where the terpene mixture apparently reduced displacement of the radioligand by Δ^9^-THC. While this may provide some evidence of terpene effects on binding, it is weak because of the small effect size.

The general lack of terpenoids effects on binding is not sufficient to completely rule out allosteric effects on function, as binding and functional modulation are separate in theory (reviewed in Lindsley et al., 2016); receptor functional modulation may not necessarily be predicted by altered binding and *vice versa*. However, neither CB1 or CB2 cAMP signaling was detectably modified by terpenes or terpene mixtures in this study. Terpenes failed to alter the efficacy (Emax) of Δ^9^-THC or 2-AG, and also showed no ability to change Δ^9^-THC potency at either CB1 or CB2. A change in potency would have been detected through the signaling assays carried out at approx. EC50 concentrations of Δ^9^-THC) (**Figures 3G** and **4G**). This approach has good sensitivity to detect potency shifts either toward Emax (i.e., increasing the potency of Δ^9^-THC) or away from Emax (decreasing the potency of Δ^9^-THC). This negative finding for signaling modulation is particularly inconsistent for β-caryophyllene, which has previous been described as a CB2 agonist with affinity in the high nanomolar range (Gertsch et al., 2008). The reason for this lack of effect is not clear, although notably our data is consistent with the recent report by Santiago et al. (2019).

Importantly, this study cannot rule out the existence of an entourage effect for terpenoids. However, in combination with Santiago et al. (2019), there *is* likely now sufficient data to rule out *direct interactions* with either cannabinoid receptor as being the mechanism by which an entourage effect is mediated, so attention must move to other types of effect. Within the endocannabinoid system, this would mean investigating the effect of terpenoids on metabolism or synthesis of the endocannabinoids.

Some researchers suggest that an entourage-related mechanism of action may not be necessary—terpenes may merely have their own biological activity, and interact functionally with the activity of Δ^9^-THC (Murataeva et al., 2016). Another mechanism which may help explain putative differences between whole cannabis and Δ^9^-THC alone is that relevant compounds may synergize functionally through different receptor targets. Such a mechanism has been suggested to explain the activity of N-acyl lipids on anandamide, *via* effects mediated by TRPV1 receptors (Smart et al., 2002; Ho et al., 2008). Other non-cannabinoid targets for terpenes have also been proposed, including the suggestion that limonene may exhibit anxiolytic-like activity *via* a GABAergic mechanism (de Almeida et al., 2012; Lima et al., 2013), although these data do not necessarily reflect direct GABA receptor effects. In another example, the terpene linalool has been put forward as a candidate NMDA receptor antagonist in a study involving both molecular and *in vivo* characterization (Elisabetsky et al., 1999). In fact, the spectrum of possible effects—including both polypharmacy (functional interactions derived from simultaneous effects of multiple drugs acting in a biological system) and polypharmacology (functional interactions derived from simultaneous effects of a drug acting at more than one target)—may help explain the entourage effect, even if this tangle of complex interactions cannot yet be unfurled by the limits of current scientific method. Finally, considering the volatility of the terpenoids (terpenoids, not cannabinoids, give cannabis its odor), it is possible that its effects may be sensory. This hypothesis also has precedent; for example, citrus terpenoids (which includes limonene, the most common naturally occurring terpenoid) have been shown to have therapeutic effects in humans, as patients who were hospitalized for depression and were exposed to citrus fragrance demonstrated improvements in Hamilton Depression Scores (Komori et al., 1995; reviewed in Russo, 2011).

It is often very difficult to distinguish between studies that support the idea of biological activity of terpenoids (including many reviewed by Russo, 2011) and studies that *specifically* address the putative entourage effect of whole cannabis, of which there are far fewer. It is worth nothing that even in human subjects, evidence is adduced against entourage—a notable instance is a study comparing the analgesic effects of pure Δ^9^-THC (dronabinol) with smoked marijuana in a rigorous (randomized, placebo-controlled, double-dummy, double-blind) clinical study. Although both groups demonstrated modest improvements in pain-related endpoints, peak changes in pain sensitivity and tolerance did not differ between marijuana and dronabinol groups (Cooper et al., 2013); indeed the author of this study has stated that she “has only ever seen evidence against the entourage effect” (Chen, 2017).

As the use of cannabis and cannabis extracts is becoming more prevalent, it remains important to investigate the potential pharmacological properties of terpenoids used in conjunction with cannabinoids. As some commentators note that “There really isn’t the science out there to support (the entourage hypothesis for whole cannabis)” (Worth, 2019), the research community must be reminded to view common opinion with some skepticism if it is not based on robust science.

## Data Availability Statement

All datasets generated for this study are included in the article/supplementary material.

## Author Contributions

DF performed experiments, contributed to data analysis, and wrote the paper. KS and MN performed experiments and contributed to data analysis. CJ contributed to the writing of the manuscript. MG gave oversight to the project, analyzed the data, and wrote the paper.

## Funding

All research costs were paid by Soma Group.

## Conflict of Interest

CJ was employed by Soma Group.

The remaining authors declare that the research was conducted in the absence of any commercial or financial relationships that could be construed as a potential conflict of interest.

The research was funded by Soma Group. Soma is a New Zealand based science-led medicinal cannabis company. While they conceived the initial concept for the study they were not involved in the final experimental design, collection, or analysis of the data or interpretation of the results.
